# Overcoming Supply Shortage for SARS-CoV-2 Detection by RT-qPCR

**DOI:** 10.3390/genes12010090

**Published:** 2021-01-13

**Authors:** Gustavo Barcelos Barra, Ticiane Henriques Santa Rita, Pedro Góes Mesquita, Rafael Henriques Jácomo, Lídia Freire Abdalla Nery

**Affiliations:** Research and Development Section, Sabin Medicina Diagnóstica, 70632-340 Brasília, Brazil; ticianehenriques@sabin.com.br (T.H.S.R.); pedro.mesquita@sabin.com.br (P.G.M.); rafaeljacomo@sabin.com.br (R.H.J.); lidia@sabin.com.br (L.F.A.N.)

**Keywords:** SARS-CoV-2, validation, RT-qPCR

## Abstract

In February 2020, our laboratory started to offer a RT-qPCR assay for the qualitative detection of severe acute respiratory syndrome coronavirus 2. A few months after the assay was released to our patients, some materials, reagents, and equipment became in short supply. Alternative protocols were necessary in order to avoid stopping testing to the population. However, the suitability of these alternatives needs to be validated before their use. Here, we investigated if saliva is a reliable alternative specimen to nasopharyngeal swabs; if 0.45% saline is a reliable alternative to guanidine hydrochloride as a collection viral transport media; the stability of SARS-COV-2 in guanidine hydrochloride and in 0.45% saline for 10 and 50 days at room temperature; and if the primers/probe concentration and thermocycling times could be reduced so as to overcome the short supply of these reagents and equipment, without a significant loss of the assay performance. We found that saliva is not an appropriated specimen for our method—nasopharyngeal swabs perform better. Saline (0.45%) and guanidine hydrochloride have a similar SARS-CoV-2 diagnostic capability as tube additives. Reliable SARS-CoV-2 RNA detection can be performed after sample storage for 10 days at room temperature (18–23 °C) in both 0.45% saline and guanidine hydrochloride. Using synthetic RNA, and decreasing the concentration of primers by five-fold and probes by 2.5-fold, changed the assay limit of detection (LOD) from 7.2 copies/reaction to 23.7 copies/reaction and the subsequent reducing of thermocycling times changed the assay LOD from 23.7 copies/reaction to 44.2 copies/reaction. However, using real clinical samples with Cq values ranging from ~12.15 to ~36.46, the results of the three tested conditions were almost identical. These alterations will not affect the vast majority of diagnostics and increase the daily testing capability in 30% and increase primers and probe stocks in 500% and 250%, respectively. Taken together, the alternative protocols described here overcome the short supply of tubes, reagents and equipment during the SARS-CoV-2 pandemic, avoiding the collapse of test offering for the population: 105,757 samples were processed, and 25,156 SARS-CoV-2 diagnostics were performed from 9 May 2020 to 30 June 2020.

## 1. Introduction

In February 2020, our laboratory started to offer an RT-qPCR assay for the qualitative detection of severe acute respiratory syndrome coronavirus 2 (SARS-CoV-2) for Brazil’s Federal District population. The validated protocol was an optimization of the assay described by the Centers for Disease Control and Prevention (CDC) [1]. A few months after the assay was released to our patients, some materials, reagents, and equipment became in short supply. Alternative protocols were necessary in order to avoid stopping testing for the population. However, the suitability of these alternatives needed to be validated before their use [2]. 

The most often used and reliable clinical specimen for SARS-CoV-2 detection is nasopharyngeal swabs samples [3]. However, acquiring a nasopharyngeal swab is not as easy as obtaining other types of specimens, such as saliva, and may result in suboptimal specimens, particularly if the specimens are obtained by inexperienced personnel. The procedure can cause coughing, increasing the risk of nosocomial spread of respiratory viruses [4]. Moreover, nasal swabs for sample collection are in short supply from our providers, and saliva could be a good candidate as alternative primary sample to mitigate these sample collection difficulties.

Additionally, we validated the sample collection in tubes containing guanidine hydrochloride. This viral transport media virtually inactivates the pathogens present in the sample and preserves the nucleic acid of the specimen [5,6,7,8], however, it this also ended up resulting in a short supply. It has been described that 0.9% saline and phosphate-buffered saline (PBS) are appropriated viral transport media for SARS-CoV-2 collection and testing during the shortage of commercials tubes containing a viral transport medium [9,10]. Saline at a lower concentration (e.g., 0.45%) may be more appropriate, but descriptions of the use of this lower concentration of saline as a viral transport media for SARS-CoV-2 detection is scarce or absent from the scientific literature.

Moreover, molecular detection methods do not require the replication of a competent virus, but the preservation of nucleic acid is essential [2]. The stability of SARS-CoV-2 or its RNA overtime in specimens collected in different tubes additives (e.g., guanidine hydrochloride and 0.45% saline) are pre-analytical knowledge gaps that can influence the accuracy of SARS-CoV-2 detection using RT-qPCR. Because of this, the ex-vivo virus stability must be investigated, especially at room temperature, in order to eliminate the need for a cold chain during the challenging period of a pandemic [11].

Furthermore, the number of samples referred to our laboratory for SARS-CoV-2 RNA detection increased substantially following the rapid spread rate of the virus, which is attributed to its ability to be transmitted before becoming symptomatic [12,13,14]. Primers and probes, as well as new qPCR thermocyclers, were in short supply at their manufacturers [15], halting us from providing more tests to our patients. Decreasing the primer/probe concentrations in the reaction and diminishing the RT-qPCR cycling times can be alternatives to restore the testing capacity, thus avoiding the collapse of test availability [16,17].

In light of the above-mentioned factors, in this study, we investigated the following questions regarding alternatives to our validated protocol for SARS-CoV-2 qualitative detection:

(a) Is saliva a reliable alternative specimen to nasopharyngeal swabs?
(b) Is 0.45% saline a reliable alternative to guanidine hydrochloride as a collection viral transport media?
(c) Can SARS-COV-2 collected in guanidine hydrochloride or in 0.45% saline be detected after 10 and 50 days of incubation at room temperature (18–23 °C)?
(d) Can the primer/probe concentration and thermocycling times be reduced to overcome the short supply of reagents and equipment without a significant loss in the RT-qPCR assay performance?

## 2. Materials and Methods 

### 2.1. Primary Samples Collection and Processing

To test if saliva is a reliable alternative specimen to nasopharyngeal swabs, 10 volunteers that tested positive for SARS-CoV-2 in our daily routine testing were invited to donate paired saliva and nasopharyngeal samples. Sample collection was performed at their residence using the appropriate protection equipment. Volunteers were asked to spit ~2.5 mL of voided saliva into a tube containing guanidine hydrochloride (Cobas^®^ PCR Media tubes from Roche Molecular Systems, Inc., Basel, Switzerland). One nasopharyngeal swab was collected and placed into another guanidine hydrochloride tube.

To test if 0.45% saline is a reliable alternative viral transport media, the 10 volunteers that tested positive for SARS-CoV-2 were invited to donate two nasopharyngeal swabs (one form each nostril), which were placed into a 0.45% saline tube (in house produced 15 mL tube with 2.5 mL of 0.45% saline) and into a guanidine hydrochloride tube, respectively. These specimens were also used to test the sample stability after 10 and 50 days of incubation at room temperature (18–23 °C). These tubes were tested for SARS-CoV-2 RNA at day 0, and were retested after 10 and 50 days, respectively.

To evaluate if the primer/probe concentrations and thermocycling times could be reduced without a loss of assay performance, a previously described synthetic SARS-CoV-2 RNA fragment was introduced directly into the RT-qPCR reactions [1]. Additionally, 105 anonymized leftover RNA samples that were positive for the virus were submitted to the three tested conditions (validated primer/probe concentrations and thermocycling conditions, named (+); decreased primer/probe concentrations and validated thermocycling conditions, named [−]; and decreased primer/probe concentrations and reduced thermocycling times, named [−] fast) in order to evaluate their effect on the Cq values.

### 2.2. Nucleic Acid Extraction

When applied, the nucleic acids were extracted from 200 μL of the primary sample using a MagNA Pure 96 Instrument (Roche Diagnostics, Basel, Switzerland) and MagNA Pure 96 DNA and Viral NA Small Volume Kit (protocol Viral NA Universal version 4.0, Roche Diagnostics, Basel, Switzerland), with elution in 100 μL of elution buffer (Roche Diagnostics, Basel, Switzerland).

### 2.3. One-Step RT-qPCR Reaction and Thermocycling Conditions

The RT-qPCR reaction (20 μL) consisted of 4 μL of LightCycler^®^ Multiplex RNA Virus Master (5×; Roche, Basel, Switzerland), 1500 nM of (or 300 nM for experiments with a reduced concentration) N1 SARS-CoV-2 primer and 375 nM (or 150 nM for experiments with ra educed concentration) probe (FAM), 80 nM of RPP30 primer and 40 nM of RPP30 probe (HEX), 0.1 μL of reverse transcriptase (200×) (Roche), 10 μL of extracted nucleic acid, and 3.34 μL of nuclease-free water. The primer and probe sequences were described elsewhere [1]. The thermocycling conditions were reverse transcription (10 min at 50 °C) and polymerase activation (3 min at 94 °C), followed by 45 cycles of 15 s at 95 °C and 30 s at 55 °C, with a total time of 85 min or a reverse transcription of 5 min at 50 °C; polymerase activation (30 s at 94 °C); and this was followed by 45 cycles of 5 s at 95 °C and 15 s at 55 °C, for a total time of 60 min. The instrument used was LightCycler 480 II.

### 2.4. Amplification Efficiency

The amplification efficiencies of each primer/probe set were investigated by testing the 10-fold dilution (1.48 × 10^8^ to 1.48 × 10^2^ copies/PCR) of the synthetic SARS-CoV-2 diagnostic RNA, followed by the evaluation of the standard curve parameters, especially its slope, from which the amplification efficiency is derived. These analyses were performed using linear regression tools available in Graphpad Prism software version 6.0 (Graphpad, Inc., La Jolla, CA, USA).

### 2.5. Determining Assays Limit of Detection

The limits of detection were calculated using the probit regression analysis of a 1:2 serial dilution (from 2.64 × 10^2^ to 4.04 × 10^−1^ copies/reaction) of the synthetic SARS-CoV-2 diagnostic RNA. Six technical replicates corresponding to each dilution point were tested in a single day (*n* = 6) and the assay response (detected or not detected) was measured. Applying the probit regression analysis to the data, a probability of detection versus concentration was returned. The target concentrations, which the assay tested positive 95% of the time (limit of detection—LOD), were estimated using Minitab version 19 (Minitab, LLC, State College, PA, USA).

## 3. Results

### 3.1. Is Saliva a Reliable Alternative Specimen to Nasopharyngeal Swabs?

**Figure 1:**
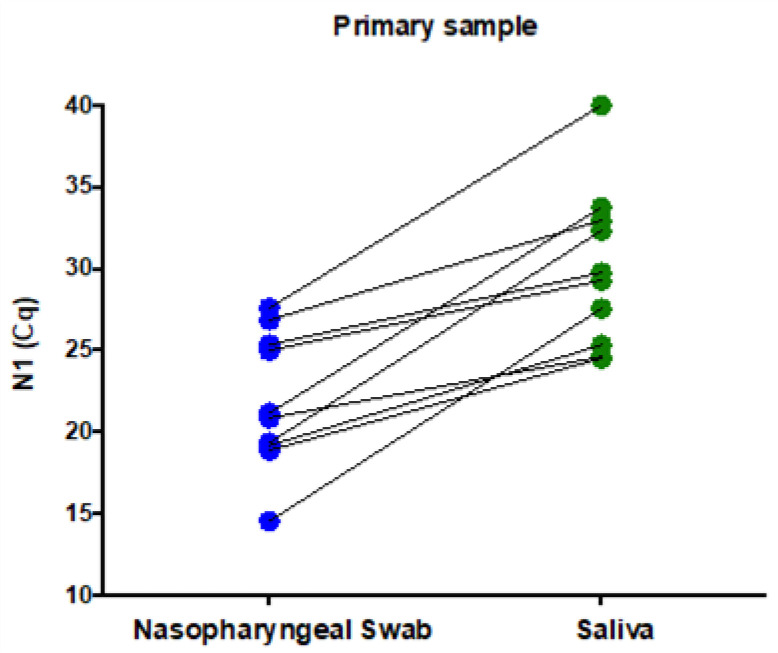
Evaluation of whether saliva (green) is a reliable alternative specimen to nasopharyngeal swabs (blue). The N1 Cq values were consistently higher in saliva compared with the paired nasopharyngeal swab sample, suggesting a decreased diagnostic capability of SARS-CoV-2 RNA in saliva.

Paired nasopharyngeal swabs and saliva samples from 10 volunteers with a recent SARS-CoV-2 diagnostic were simultaneously submitted for a new SARS-CoV-2 detection test in order to evaluate the virus RNA detection in saliva. The median (min–max) N1 Cq values were 21.01 (14.53–27.59) for nasopharyngeal swabs and 29.51 (24.50–40) for saliva (Figure 1), suggesting a lower diagnostic capability of SARS-CoV-2 in saliva.

### 3.2. Is 0.45% Saline a Reliable Alternative Collection Viral Transport Media to Guanidine Hydrochloride?

**Figure 2:**
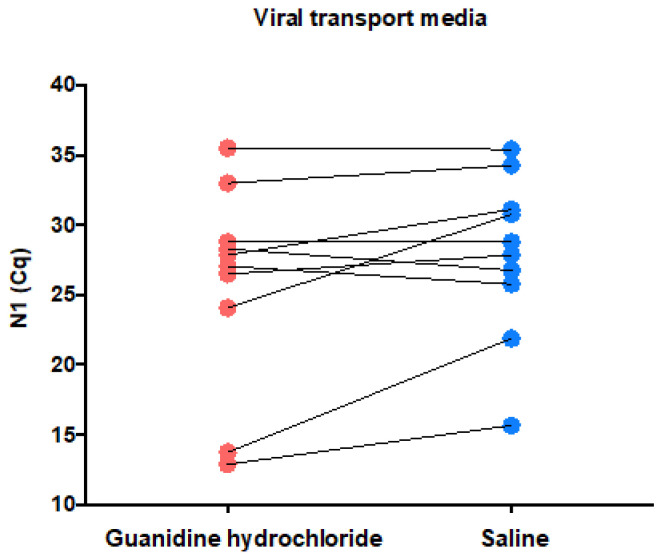
Evaluation of whether 0.45% saline (blue) is a reliable alternative viral transport media to guanidine hydrochloride (red). The N1 Cq values were analogous or slightly decrease in 0.45% saline compared with the paired guanidine hydrochloride sample, suggesting an acceptable diagnostic capability of SARS-CoV-2 in 0.45% saline.

Paired samples from the 10 volunteers with a recent SARS-CoV-2 diagnosis collected in both guanidine hydrochloride and 0.45% saline as a viral transport media were simultaneously submitted to a new SARS-CoV2 detection test in order to evaluate the virus detection in 0.45% saline. The median (min–max) N1 Cq values were 27.46 (12.88–35.52) for guanidine hydrochloride and 28.33 (15.66–35.40) for 0.45% saline (Figure 2), suggesting an acceptable and conformable diagnostic capability of SARS-CoV-2 using 0.45% saline as a viral transport media.

### 3.3. Can SARS-COV-2 Collected in Guanidine Hydrochloride or in 0.45% Saline Be Detected after 10 and 50 Days of Incubation at Room Temperature (18–23 °C)?

**Figure 3:**
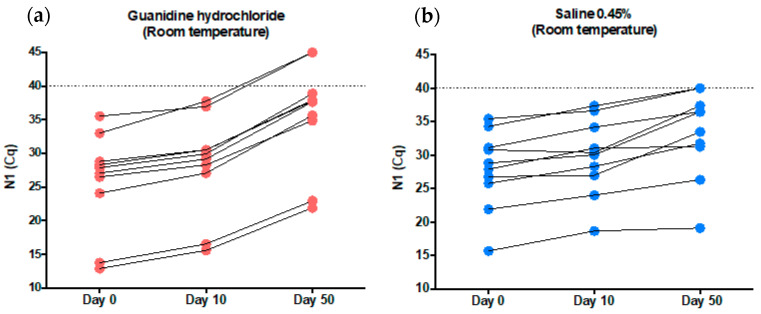
Evaluation of the SARS-CoV-2 detection capability in guanidine hydrochloride (red) (**a**) and in 0.45% saline (blue) (**b**) after 10 and 50 days of incubation at room temperature (Nasopharyngeal samples). The N1 Cq values were slightly increased on day 10 versus day 0 for both tubes additives suggesting that a reliable SARS-CoV-2 detection can be performed for 10 days at room temperature after sample collection. However, the N1 Cq values were higher on day 50 versus day 0 for both tubes’ additives, indicating that after 50 days at room temperature, the diagnostic capability is affected, especially in guanidine hydrochloride. In summary, SARS-CoV-2 diagnostics can reliably be performed in both 0.45% saline and guanidine hydrochloride after sample storage for 10 days at room temperature, but for extended periods (e.g., 50 days), the detection seems to be more reliable in 0.45% saline. The dashed line is the Cq value cut-off value and a Cq of 45 was arbitrarily attributed to samples where SARS-CoV-2 was not detected.

The same samples used in the previous experiment were stored for 10 day and then for 50 days at room temperature before been resubmitted to SARS-CoV-2 RNA detection. In guanidine hydrochloride, the median (min-max) N1 Cq values were 29.53 (15.58–37.75) for day 10 and 37.71 (21.90—not detected) for day 50 (reference: day 0–27.46 (12.88–35.52)). In 0.45% saline, the median (min–max) N1 Cq values were 30.20 (18.64–37.34) for day 10 and 34.96 (19.08–40) for day 50 (reference: day 0–28.33 (15.66–35.40)). These results suggest a slightly diminished SARS-CoV-2 diagnostic capability after 10 days and a decreased SARS-CoV-2 diagnostic capability after 50 days of sample collection in both the guanidine hydrochloride and 0.45% saline tubes. The decrease in the diagnostic capability after 50 days appears to be more pronounced in guanidine hydrochloride, suggesting less stability for SARS-CoV-2 RNA in this additive (Figure 3). In summary, SARS-CoV-2 diagnostics can reliably be performed after sample storage for 10 days at room temperature in both 0.45% saline and guanidine hydrochloride, but for extended periods (e.g., 50 days) SARS-CoV-2 detection seems to be more reliable in 0.45% saline.

### 3.4. Can the Primer/Probe Concentrations and Thermocycling Time Be Reduced to SARS-COV2 Testing Capability without Loss of Assay Performance

In the first version of our method, The N1 primer and probe concentrations per reaction were 1500 nM and 375 nM, respectively (named [+]). Here, we tested if the primer and concentration could be reduced to 300 nM and 150 nM per reaction, respectively (named [−]). After that, we investigated if the thermocycling time could be reduced from reverse transcription of 10 min at 50 °C, polymerase activation of 3 min at 94 °C, followed by 45 cycles of 15 s at 95 °C and 30 s at 55 °C, for a total time of 85 min, to reverse transcription of 5 min at 50 °C, polymerase activation of 30 s at 94 °C, followed by 45 cycles of 5 s at 95 °C and 15 s at 55 °C, for a total time of 60 min (named [−] fast).

**Figure 4:**
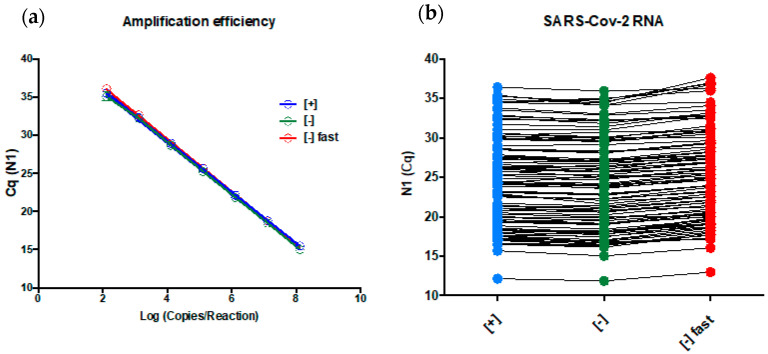
(**a**) Amplification efficiencies and (**b**) Cq values observed for 105 SARS-CoV-2 positive samples. The values for both parameters for [+] (blue), [−] (green), and [−] fast (red) were almost identical, suggesting that the three tested conditions did not affect the diagnostic capability of the N1 assay. A three-fold decrease in the limit of detection (LOD) was observed for [−] compared with [+] (7.3 versus 23.7 copies/reaction), and a six-fold decrease in the LOD was observed for [−] compared with [−] fast (7.3 versus 44.2 copies/reaction) using synthetic SARS-CoC-2 RNA and probit regression, suggesting that the differences between the tested conditions will be noted only on samples with a very low viral load.

The PCR amplification efficiencies using the SARS-CoV-2 diagnostic RNA (described in [1]) were 98.5% (y = −3.357x + 42.74) for [+], 97.5 % (y = −3.383x + 42.52) for [−], and 97.5% (y = −3.383x + 43.35) for [−] fast (Figure 4a). The probit regression analysis showed a LOD of 7.3 (95% CI 5.21–18.84) copies/reaction for [+], 23.7 (95% CI 16.1–57) copies/reaction for [−] (3.2-fold decrease versus [+]), and 44.2 (95% CI 30.2–99.4) copies/reaction for [−] fast (6-fold decrease versus [+]). Then, we submitted 105 real clinical nasopharyngeal samples positive for SARS-CoV-2 RNA collected in 0.45% saline to the three tested conditions, and they presented almost identical results (Figure 4b). The median (min–max) N1 Cq values were 24.21 (12.15–36.46) for [+], 23.87 (11.88–35.98) for [−], and 24.92 (12.98–37,65) for [−] fast.

## 4. Discussion

Here, we investigated the preanalytical and analytical parameters that can affect SARS-CoV-2 RNA using a CDC N1-based assay, and also tested some alternatives to our validated protocol in order to overcome the short supply of tubes, reagents, and equipment during pandemics, avoiding halting testing for the population. The most appropriate specimen and viral transport media for SARS-CoV-2 detection were evaluated. The ex-vivo SARS-CoV-2 RNA stability at room temperature was also determined. Additionally, we investigated whether the primer/probe concentrations in the reaction and the thermocycling times could be reduced without compromising the assay performance. 

First, we found that our assay performs better in nasopharyngeal swabs compared with saliva. Paired specimens were submitted to an identical process, involving RNA extraction of 200 uL of each type of sample, which were collected in tubes containing guanidine hydrochloride, but the saliva showed consistently higher Cq N1 values compared with the nasopharyngeal swabs (median Cq values of 21.01 versus 29.51). Qualitatively, no difference was observed between the tested specimens, but after observing the Cq values, we can conclude that the saliva collected using the drooling technique was not an appropriate primary sample for our assay because of the presumed decrease in the assay sensibility and diagnostic capability in samples with a low viral load. Collecting only oral fluids instead of mucous secretions from the oropharynx or lower respiratory tract may have contributed to the overall higher Cq values found saliva. Indeed, overall higher Cq values for saliva were overserved in one study [18], but not in another [19]. A systematic review concluded that saliva is a reliable sample for SARS-CoV-2 detection [20]. Our results highlight that the validation of the primary sample locally for each laboratory is important in order to achieve the best sensitivity and specificity for the SARS-CoV-2 detection method, as we found that the nasopharyngeal swab performed better in our study.

Next, we investigated if 0.45% saline is a reliable collection viral transport media compared to the validated method of guanidine hydrochloride. The median Cq values were slightly higher for 0.45% saline (28.33) versus guanidine hydrochloride (27.46). Some paired samples presented higher Cq values in 0.45% saline and others in guanidine hydrochloride, suggesting a random effect over the amount of material that could be secondary to the nasopharyngeal sampling. We conclude that 0.45% saline and guanidine hydrochloride have a similar diagnostic capability. The validation of 0.9% saline and PBS as an alternative transport medium for SARS-CoV-2 testing has been done by others [9,10]. The downside of 0.45% saline use instead of guanidine hydrochloride is that the former does not inactivate the microorganisms that may be present in the sample, and extra cautious should be applied during the sample measurement [7]. To our knowledge, this is the first description of use of 0.45% saline as a viral transport media for SARS-CoV-2 detection. Saline with a lower concentration is desirable for extraction-free RT-qPCR, because 0.9% saline and PBS are compatible with SARS-CoV-2 direct RT-qPCR only at lower input volumes [21].

The observed stability of SARS-CoV-2 RNA in both 0.45% saline and guanidine hydrochloride was high at room temperature. The virus could be reliably detected for 10 days after sample collection (median Cq values of 27.46 on day 0 versus 29.53 on day 10 for guanidine hydrochloride, and 28.33 on day 0 versus 30.20 on day 10 for 0.45% saline). The median N1 Cq value fluctuation was 1.87 in 0.45% saline and 2.07 in guanidine hydrochloride. These two tested tube additives had differences regarding the state of the SARS-CoV-2 RNA inside them. In the 0.45% saline tubes, it is expected that SARS-CoV-2 is intact, protected by its envelope and membrane proteins. On the other hand, SARS-CoV-2 RNA is unprotected in guanidine hydrochloride tubes because of its chaotropic effect, which degrades the envelope and membrane of viruses [5,6,7,8]. We conclude that SARS-CoV-2 detection is highly reliable in both samples, at least for 10 days. Additional time can negatively affect the diagnostic capabilities, especially in guanidine hydrochloride tubes, where the virus RNA is unprotected (see Figure 3a, b for 50-day results). 

The stability of SARS-CoV-2 in phosphate-buffered saline at room temperature has been studied elsewhere; for samples with high viral loads (10,000–50,000 copies per mL), the median N1 Cq values fluctuation was less than 1 though 28 days. For samples with low viral loads, the reduction in positivity began at day 7, and by day 28, 0% of samples were detected using N1 [11]. These findings corroborate our results indicating reliable stability of SARS-CoV-2 at room temperatures for 7–10 days in different viral transport media. To our knowledge, this is the first description of SARS-CoV-2 stability in 0.45% saline and guanidine hydrochloride for diagnostic purposes.

The primer/probe concentrations used in the validated protocol are high, and increase the assay sensitivity [1]. Scaling down the primer/probe concentration to legacy levels would allow us to perform more diagnostic tests with the same amount of material. The challenge is to perform this scaling down without a significant loss of sensitivity. It is important to highlight that many oligo manufactures experienced undesirable SARV-CoV-2 target contamination during the manufacturing, which lead to a short or delayed supply of these reagents [22]. So, as many assays as possible must be performed with approved uncontaminated primers lots. Using synthetic SARS-COV-2 diagnostic RNA, the reduction in the N1 primer concentration from 1500 nM to 300 nM (five-fold or 500%) and the probe from 375 nM to 150 nM (2.5-fold or 250%) did not affect the PCR amplification efficiency of the assay, but a 3.2-fold decrease in LOD was observed (7.3 versus 23.7 copies/reaction). However, it is expected that the diagnostic capability of the clinical samples with viral loads higher than 23.7 copies/reaction is not affected, as observed in Figure 4b, where [+] (high amount of oligos) and [−] (less amount of oligos) showed very similar N1 Cq values on extracted RNA samples that were tested positive for SARS-CoV-2 (median Cq values of 24.21 for [+] versus 23.87 for [−]). We concluded that a decrease in the primer/probe concentration will not affect the vast majority of the diagnostics, only those with viral loads between 7.2 copies/reaction (~722 copies/mL of viral transport media) and 23.7 copies/reaction (~2370 copies/mL of viral transport media).

Reducing the thermocycling time is an alternative to increase the number of tests that could be performed per day, as the new thermocycler was under stock depletion from the manufactures. Decreasing the reaction time from 1 h and 25 min to 1 h would increase the testing capability by 30%, eliminating our necessity of new thermocyclers. Using synthetic SARS-CoV-2 diagnostic RNA, the reduction in the thermocycling time as described above did not affect the PCR amplification efficiency of the N1 assay, but did decrease the assay LOD to 44.2 copies/reaction (six-fold compared with the original LOD). The same deliberation used above for the primer concentration can be used here (median Cq values of 23.87 for [−] versus 24.92 for [−] fast). We concluded that reducing the thermocycling time will not affect the vast majority of diagnostics, only those with viral loads between 7.2 copies/reaction (~722 copies/mL viral transport media) and 44.2 copies/reaction (~4420 copies/mL of viral transport media). 

It is expected that limit of detection matters and low viral load infections will be missed [23]. However, these modifications increased our daily testing capability by 30% and our reagent stock by 500% for primers and 250% for probes, which have a more profound effect on combating the pandemic in Brazil’s Federal District and other Brazilian regions that send their samples to us. Using real clinical samples with Cq value ranging from ~12.15 to ~36.46, the results of the three tested conditions were almost identical. This observation corroborates the amplification efficiency experiment, where the assay presented a linear response similar to this range (Figure 4a). Indeed, some laboratories use a Cq cut-off value of 35–36 [24], which means that reactions that returns Cq higher that these values are judge as negative or indeterminate results. Our assay presented a linear response until Cq 36 suggesting the higher Cq values can be detected. Taken together, the above-described evidences favor our statement that primer/probe concentration and thermocycling modifications will not affect the vast majority of diagnostics.

## 5. Conclusions

In conclusion, (a) saliva is not an appropriate specimen for our method, nasopharyngeal swabs perform better; (b) saline (0.45%) and guanidine hydrochloride have similar SARS-CoV-2 diagnostic capability as tube additives; (c) reliable SARS-Cov-2 RNA detection can be performed after sample storage for 10 days at room temperature using 0.45% saline and guanidine hydrochloride as the collection medium; (d) decreasing the primers and probe concentration increases the LOD of the assay by 3.3-fold; (e) reducing the thermocycling time increases the LOD by 6.1-fold; and both alterations (d and e) will not affect the vast majority of diagnostics, but will increase our daily testing capability and our primer/probe stocks. Using 0.45% saline as a viral transport media and reducing primer and thermocycling conditions together with using the optimized direct PCR method (in publication), 105,757 samples were processed, and 25,156 SARS-CoV-2 diagnostics were performed from 9 May 2020 to 30 June 2020 by our laboratory.

## Data Availability

The data presented in this study are contained within the article.

## References

[1] Barra G.B., Santa Rita T.H., Mesquita P.G., Jácomo R.H., Nery L.F.A. (2020). Analytical Sensitivity and Specificity of Two RT-QPCR Protocols for SARS-CoV-2 Detection Performed in an Automated Workflow. Genes.

[2] Druce J., Garcia K., Tran T., Papadakis G., Birch C. (2012). Evaluation of Swabs, Transport Media, and Specimen Transport Conditions for Optimal Detection of Viruses by PCR. J. Clin. Microbiol..

[3] Wang W., Xu Y., Gao R., Lu R., Han K., Wu G., Tan W. (2020). Detection of SARS-CoV-2 in Different Types of Clinical Specimens. JAMA.

[4] Tellier R. (2006). Review of Aerosol Transmission of Influenza A Virus. Emerg. Infect. Dis..

[5] Kravchenko A.V., Chetverina E.V., Chetverin A.B. (2006). Retention of nucleic acid integrity in guanidine thiocyanate lysates of whole blood. Bioorg. Khim..

[6] Tang Y.-W., Schmitz J.E., Persing D.H., Stratton C.W. (2020). Laboratory Diagnosis of COVID-19: Current Issues and Challenges. J. Clin. Microbiol..

[7] Welch S.R., Davies K.A., Buczkowski H., Hettiarachchi N., Green N., Arnold U., Jones M., Hannah M.J., Evans R., Burton C. (2020). Analysis of Inactivation of SARS-CoV-2 by Specimen Transport Media, Nucleic Acid Extraction Reagents, Detergents, and Fixatives. J. Clin. Microbiol..

[8] Blow J.A., Dohm D.J., Negley D.L., Mores C.N. (2004). Virus Inactivation by Nucleic Acid Extraction Reagents. J. Virol. Methods.

[9] Radbel J., Jagpal S., Roy J., Brooks A., Tischfield J., Sheldon M., Bixby C., Witt D., Gennaro M.L., Horton D.B. (2020). Detection of Severe Acute Respiratory Syndrome Coronavirus 2 (SARS-CoV-2) Is Comparable in Clinical Samples Preserved in Saline or Viral Transport Medium. J. Mol. Diagn..

[10] Rodino K.G., Espy M.J., Buckwalter S.P., Walchak R.C., Germer J.J., Fernholz E., Boerger A., Schuetz A.N., Yao J.D., Binnicker M.J. (2020). Evaluation of Saline, Phosphate-Buffered Saline, and Minimum Essential Medium as Potential Alternatives to Viral Transport Media for SARS-CoV-2 Testing. J. Clin. Microbiol..

[11] Perchetti G.A., Huang M.-L., Peddu V., Jerome K.R., Greninger A.L. (2020). Stability of SARS-CoV-2 in Phosphate-Buffered Saline for Molecular Detection. J. Clin. Microbiol..

[12] Streeck H., Schulte B., Kümmerer B.M., Richter E., Höller T., Fuhrmann C., Bartok E., Dolscheid-Pommerich R., Berger M., Wessendorf L. (2020). Infection Fatality Rate of SARS-CoV2 in a Super-Spreading Event in Germany. Nat. Commun..

[13] Mizumoto K., Kagaya K., Zarebski A., Chowell G. (2020). Estimating the Asymptomatic Proportion of Coronavirus Disease 2019 (COVID-19) Cases on Board the Diamond Princess Cruise Ship, Yokohama, Japan, 2020. Eurosurveillance.

[14] Lai C.-C., Liu Y.H., Wang C.-Y., Wang Y.-H., Hsueh S.-C., Yen M.-Y., Ko W.-C., Hsueh P.-R. (2020). Asymptomatic Carrier State, Acute Respiratory Disease, and Pneumonia Due to Severe Acute Respiratory Syndrome Coronavirus 2 (SARS-CoV-2): Facts and Myths. J. Microbiol. Immunol. Infect..

[15] Sahajpal N.S., Mondal A.K., Njau A., Ananth S., Jones K., Ahluwalia P.K., Ahluwalia M., Jilani Y., Chaubey A., Hegde M. (2020). Effective Optimization of SARS-CoV-2 Laboratory Testing Variables in an Era of Supply Chain Constraints. Future Microbiol..

[16] Mikeska T., Dobrovic A. (2009). Validation of a Primer Optimisation Matrix to Improve the Performance of Reverse Transcription—Quantitative Real-Time PCR Assays. BMC Res. Notes.

[17] Bustin S.A., Nolan T. (2020). RT-QPCR Testing of SARS-CoV-2: A Primer. Int. J. Mol. Sci..

[18] Procop G.W., Shrestha N.K., Vogel S., Van Sickle K., Harrington S., Rhoads D.D., Rubin B.P., Terpeluk P. (2020). A Direct Comparison of Enhanced Saliva to Nasopharyngeal Swab for the Detection of SARS-CoV-2 in Symptomatic Patients. J. Clin. Microbiol..

[19] Wyllie A.L., Fournier J., Casanovas-Massana A., Campbell M., Tokuyama M., Vijayakumar P., Warren J.L., Geng B., Muenker M.C., Moore A.J. (2020). Saliva or Nasopharyngeal Swab Specimens for Detection of SARS-CoV-2. N. Engl. J. Med..

[20] Fakheran O., Dehghannejad M., Khademi A. (2020). Saliva as a Diagnostic Specimen for Detection of SARS-CoV-2 in Suspected Patients: A Scoping Review. Infect. Dis. Poverty.

[21] Smyrlaki I., Ekman M., Lentini A., Rufino de Sousa N., Papanicolaou N., Vondracek M., Aarum J., Safari H., Muradrasoli S., Rothfuchs A.G. (2020). Massive and Rapid COVID-19 Testing Is Feasible by Extraction-Free SARS-CoV-2 RT-PCR. Nat. Commun..

[22] Wang C.Y.T., Buckley C., Bletchly C., Harris P., Whiley D. (2020). Contamination of SARS-CoV-2 RT-PCR Probes at the Oligonucleotide Manufacturer. Pathology.

[23] Arnaout R., Lee R.A., Lee G.R., Callahan C., Yen C.F., Smith K.P., Arora R., Kirby J.E. (2020). SARS-CoV2 Testing: The Limit of Detection Matters. bioRxiv.

[24] Chang M.C., Hur J., Park D. (2020). Interpreting the COVID-19 Test Results: A Guide for Physiatrists. Am. J. Phys. Med. Rehabil..

